# Dataset on the effect of sodium sources on the morphology, crystallite size and carbon content of NaTi_2_(po_4_)_3_/c composite prepared by an in situ process

**DOI:** 10.1016/j.dib.2020.105871

**Published:** 2020-06-18

**Authors:** Yiming Ding, Shaojie Zhang, Maolong Li, Chao Ma, Zixiang Zhang, Wutao Mao, Keyan Bao

**Affiliations:** School of Chemical and Environmental Engineering, Jiangsu University of Technology, Changzhou, 213001, China

**Keywords:** In situ process, Nati2(po4)3/c composite, Crystallite size, carbon content

## Abstract

The data presented in this manuscript showed the effects of the sodium sources on the morphology, crystallite size and carbon content of NaTi_2_(PO_4_)_3_/C composite obtained after heat treatment under N_2_ atmosphere of the as-spraydried powders. The morphology, crystalline size and carbon content of three products with different sodium sources were investigated.

The data are related to “Synthesis of NaTi_2_(PO_4_)_3_@C microspheres by an in situ process and their electrochemical properties” (Mao et al.,2020).

Specifications table

**Table:** 

Subject	Chemistry
Specific subject area	Inorganic chemistry
Type of data	Figure, Table
How data were acquired	FE-SEM (SEM, S-3400 N), thermal analyzer(STA25000), Laser Particle Size Analyser (Winner 2000E)
Data format	Raw, Analyzed
Parameters for data collection	Carbon precursor
Description of data collection	Morphology, crystallite size, carbon content of NaTi_2_(PO_4_)_3_/C composite
Data source location	Changzhou, China.
Data accessibility	Data included in this article
Related research article	Wutao Mao, Shaojie Zhang, Junli Pan, Yiming Ding, Chao Ma, Maolong Li, Fengpu Cao, Zhiguo Hou*, Keyan Bao*, Yitai Qian, Synthesis of NaTi_2_(PO_4_)_3_@C microspheres by an in situ process and their electrochemical properties, Journal of alloys and compounds, doi.org/10.1016/j.jallcom.2020.155300

## Value of the data

• These data provide a better understanding for the appropriate sodium sources with different carbon chains to prepare NaTi_2_(PO_4_)_3_/C composite by an in situ process.
• These data can be useful to those who fabricate the NaTi_2_(PO_4_)_3_/C composite microspheres with suitable carbon content and suitable particle size.
• These data can be applied for the synthesis of appropriate other inorganic compound/C composite.

## 1 Data description

**Fig. 1:**
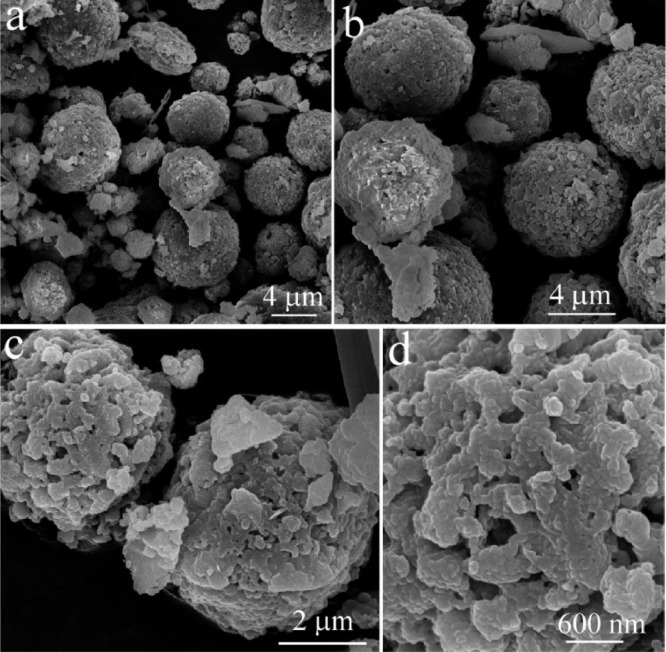
FE-SEM images of NaTi_2_(PO_4_)_3_/C composite prepared with disodium EDTA as the sodium source.

**Fig. 2:**
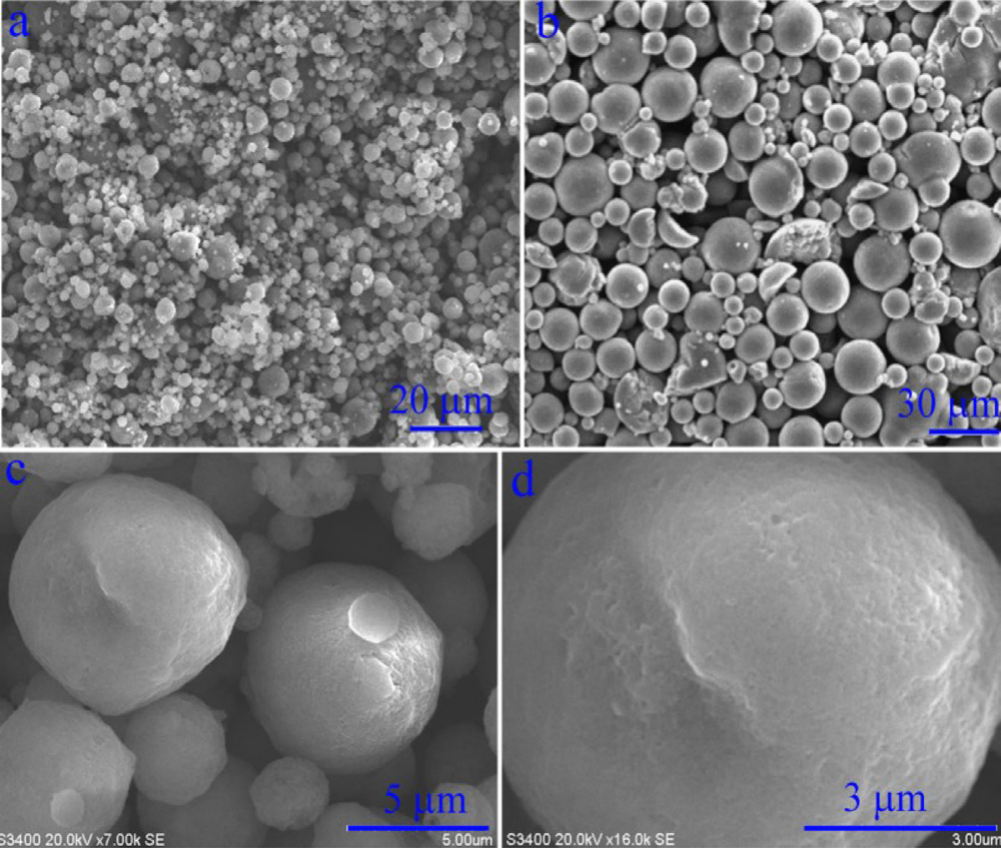
FE-SEM images of NaTi_2_(PO_4_)_3_/C composite prepared with trisodium citrate dihydrate as the sodium source.

**Fig. 3:**
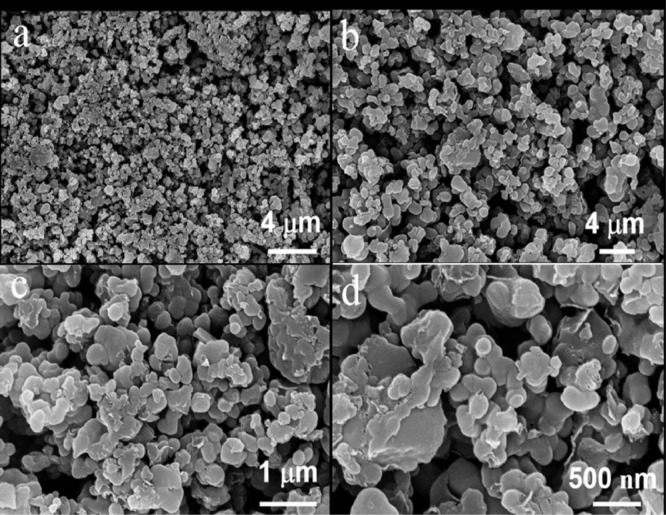
FE-SEM images of NaTi_2_(PO_4_)_3_/C composite prepared with sodium benzoate as the sodium source.

**Fig. 4:**
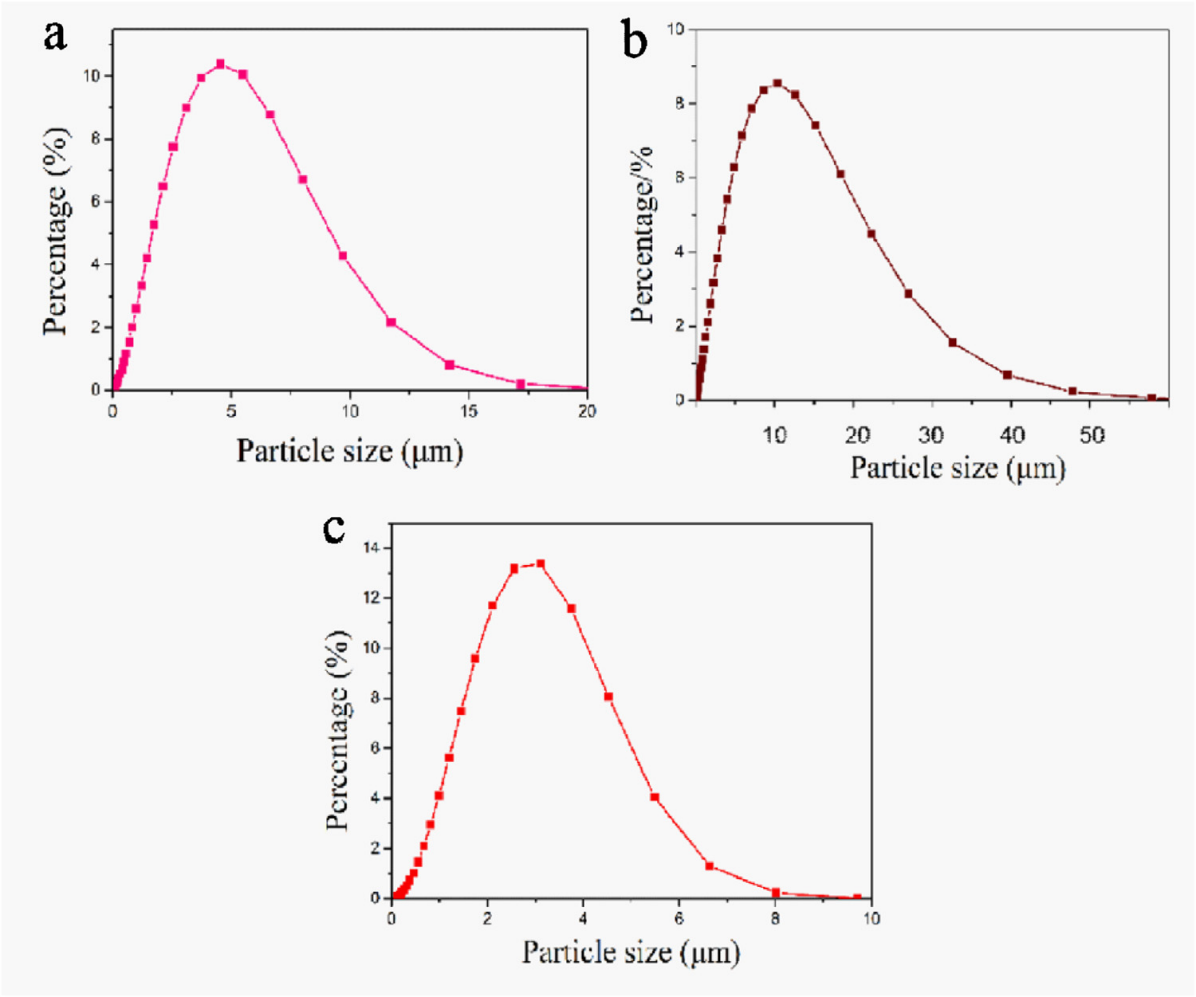
crystallite size distribution of NaTi_2_(PO_4_)_3_/C composite prepared with (a) disodium EDTA (b) trisodium citrate dihydrate (c) sodium benzoate as the sodium source.

**Fig. 5:**
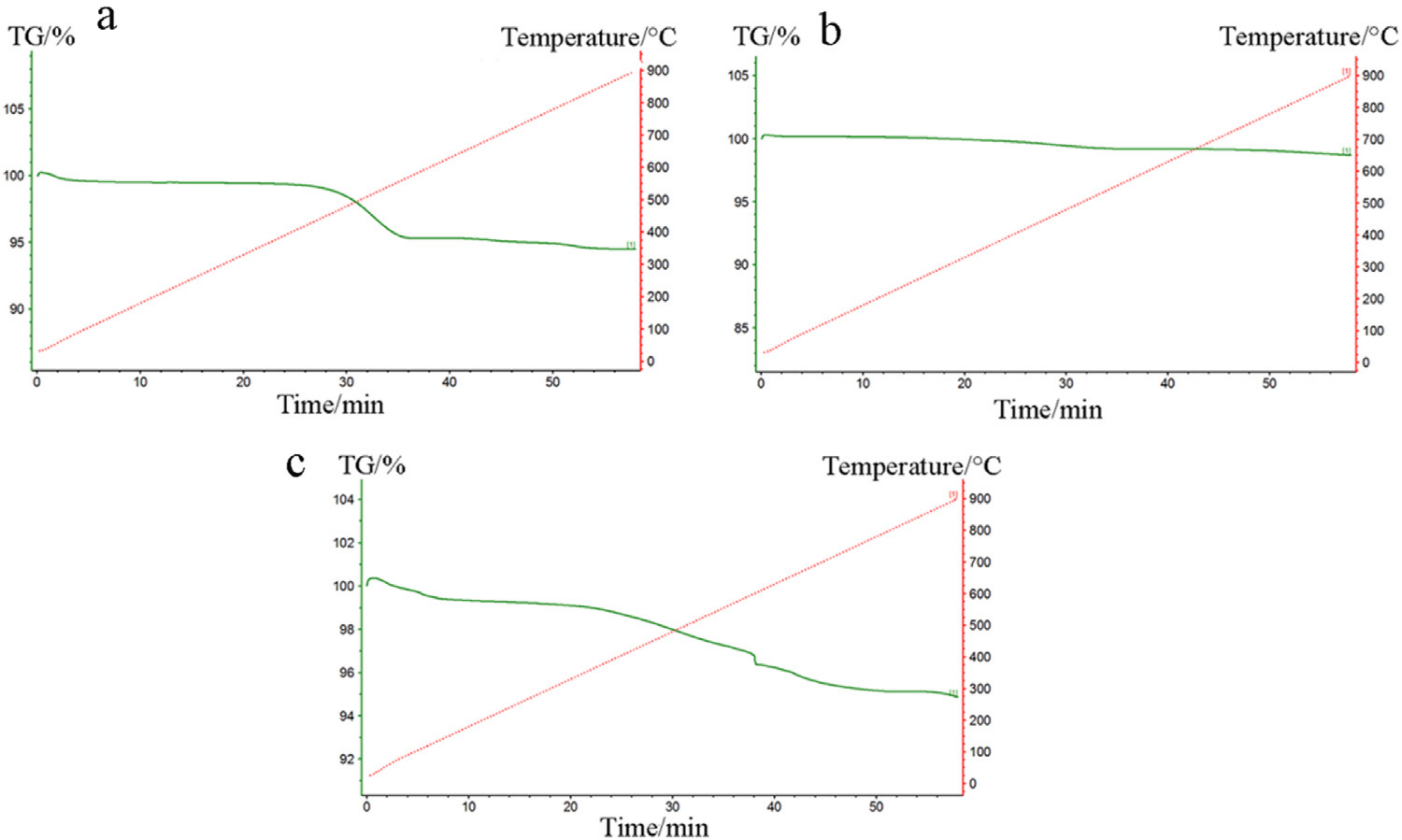
TG curves of NaTi_2_(PO_4_)_3_/C composite prepared with (a) disodium EDTA (b) trisodium citrate dihydrate (c) sodium benzoate as the sodium source.

The data exhibited in this manuscript show the effects of the sodium source on the morphology, crystallite size, and carbon content of the NaTi_2_(PO_4_)_3_/C composite. Fig. 1 shows the morphology of as-sprayed NaTi_2_(PO_4_)_3_/C composite microspheres with disodium EDTA as starting material. Fig. 2 shows the morphology of as-sprayed NaTi_2_(PO_4_)_3_/C composite microspheres with trisodium citrate dihydrate as starting material. Fig. 3 shows the morphology of as-sprayed NaTi_2_(PO_4_)_3_/C composite particles with sodium benzoate as starting material. Fig. 4 shows crystallite size distribution of the three products prepared from disodium EDTA, trisodium citrate dihydrate and sodium benzoate respectively. Fig. 5 shows carbon content of the three products prepared from disodium EDTA, trisodium citrate dihydrate and sodium benzoate respectively.

## 2 Experimental design, materials, and methods

In this experiment, NaTi_2_(PO_4_)_3_@C microsphere particles were prepared by spray drying and heat treatment after simple ball milling. A certain amount of sodium source such as disodium EDTA, trisodium citrate dihydrate and sodium benzoate, tetraethyl titanate (C_8_H_20_O_4_Ti, 0.02 mol, 4.65 g) and NH_4_H_2_PO_4_ (0.03 mol, 3.45 g) were added along with 10 mL of water to a 100 mL stainless steel ball mill jar. It is necessary to note that the molar ratio of Na^+^, tetraethyl titanateand, and NH_4_H_2_PO_4_ must be 1:2:3. The mixture was processed in a planetary ball mill (KQM-Z/B) by rapid grinding for 5 h at 500 rpm . A spray dryer (HF-6000F) was used to produce the NaTi_2_(PO_4_)_3_/C composite precursor. The slurry mixture was injected through the peristaltic pump at a speed of 25 rpm and atomized by the nozzle to produce tiny spherical droplets. The precursors were obtained by injecting the droplets downward into a heating chamber at 220 °C. The final products were prepared by calcining the obtained precursor with Na^+^:Ti^4+^:PO_4_^3−^ molar ratio of 1:2:3 at 800 °C for 10 h in N_2_. The amounts of the three sodium sources corresponding to three products are listed in Table 1.

**Table 1:** the amounts of the three sodium sources corresponding to three products.

	disodium EDTA	Trisodium citrate dihydrate	sodium benzoate
NaTi_2_(PO_4_)_3_/C No.1	0.005 mol, 1.68 g	0	0
NaTi_2_(PO_4_)_3_/C No.2	0	0.01/3 mol, 0.98 g	0
NaTi_2_(PO_4_)_3_/C No.3	0	0	0.01 mol, 1.44 g

The morphologies of the three products synthesized by using different sodium sources are shown in Fig. 1–3. Fig. 1 shows morphology of the NaTi_2_(PO_4_)_3_/C composite with disodium EDTA as the sodium source in which the spherical structure can be observed. Fig. 1a and Fig. 1b shows that the surfaces of these spherical structures contain many nanoparticles, and the diameters of these spherical structures are approximately 3–8 μm. Fig. 1c and Fig. 1d show that the surfaces of the microspheres were composed of irregular particles with size of 300–500 nm, and the surface of this composite microsphere was rough. Fig. 1 also show that there may be rich mesopores and macropores inter the microsphere.

Fig. 2 shows morphology of the NaTi_2_(PO_4_)_3_/C composite with trisodium citrate dihydrate as the sodium source in which the spherical structure can be observed. Fig. 2b shows the diameters of microspheres are but 5–20 μm. Fig. 2c and Fig. 2d shows these microspheres have bright and clean surfaces.

Fig. 3 shows morphology of the NaTi_2_(PO_4_)_3_/C composite with sodium benzoate dihydrate as the sodium source in which no microspheres and irregular bulks structure can be observed. Fig. 3 shows that irregular particles with diameters of 2–5 μm pile up together incompactly.

Fig. 4 shows crystallite size distribution of NaTi_2_(PO_4_)_3_/C composite are made from disodium EDTA, trisodium citrate dihydrate and sodium benzoate. Fig. 4a shows the particles sizes of NaTi_2_(PO_4_)_3_/C with disodium EDTA as the sodium source are 3–8 μm, and the D50 value of this product is 3.3 μm. Fig. 4b shows the particles sizes of NaTi_2_(PO_4_)_3_/C with trisodium citrate dihydrate as the sodium source are 5–20 μm, and the D50 value of this product is 13.7 μm. Fig. 4c shows the particles sizes of NaTi_2_(PO_4_)_3_/C with trisodium citrate dihydrate as the sodium source are 2–5 μm, and the D50 value of this product is 2.2 μm.

From the TG curves of these tree products as shown in Fig. 5, the carbon content can be calculate as 4.95%, 1.4%, 4.11% for NaTi_2_(PO_4_)_3_/C composite prepared from disodium EDTA, trisodium citrate dihydrate, sodium benzoate respectively.

## Declaration of Competing Interest

The authors declare that they have no known competing financial interests or personal relationships that could have appeared to influence the work reported in this paper.

## Appendix Supplementary materials

Image, application 1

Image, application 2

Image, application 3

