# Supplementary figures and images for: Dataset on the effect of sodium sources on the morphology, crystallite size and carbon content of NaTi2(po4)3/c composite prepared by an in situ process
Source: Data Brief. 2020 Jun 18;31:105871. doi: 10.1016/j.dib.2020.105871 (PMC7334292; doi:10.1016/j.dib.2020.105871)

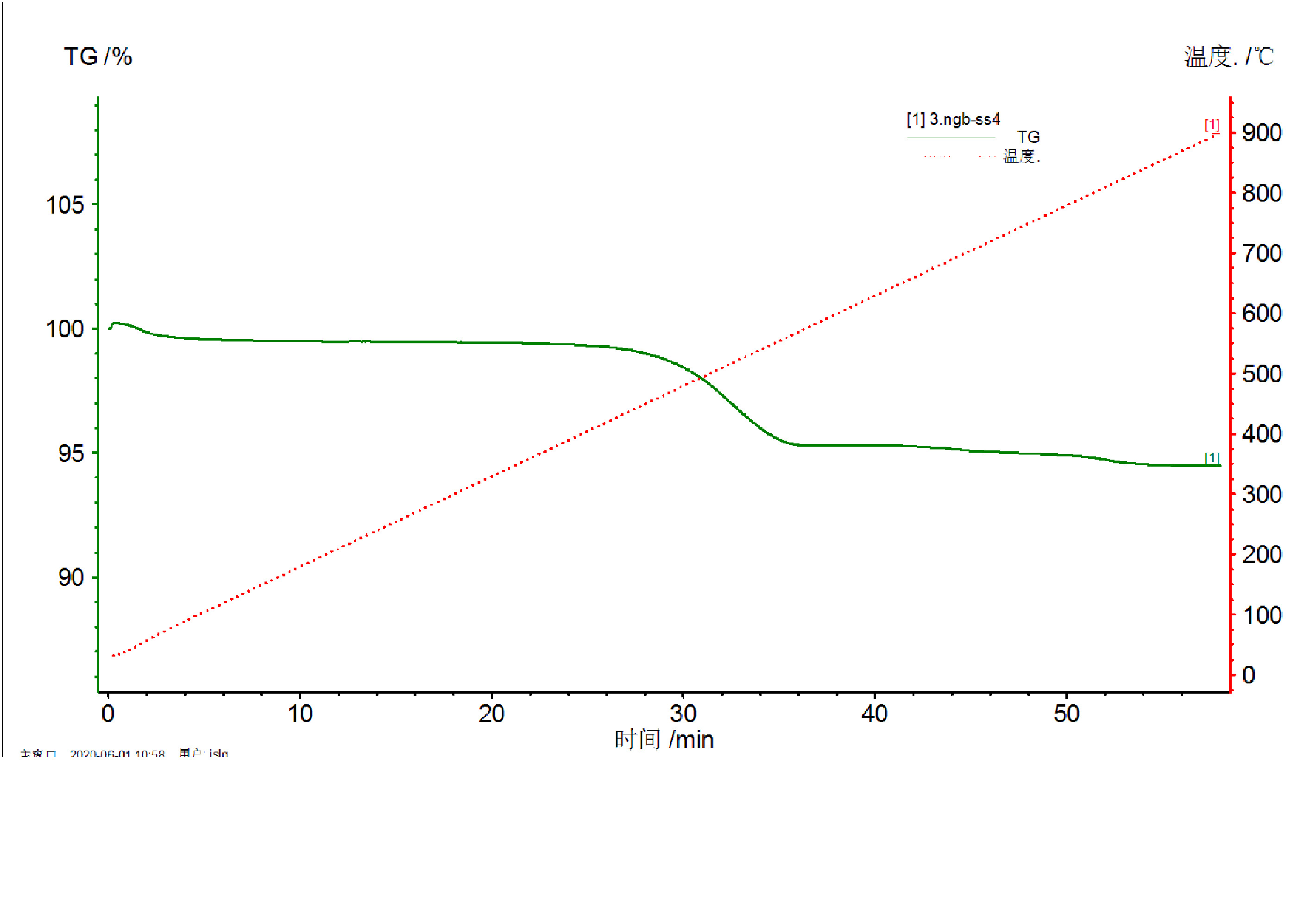

Supplement: Supplementary file 1 [file mmc1.jpg]

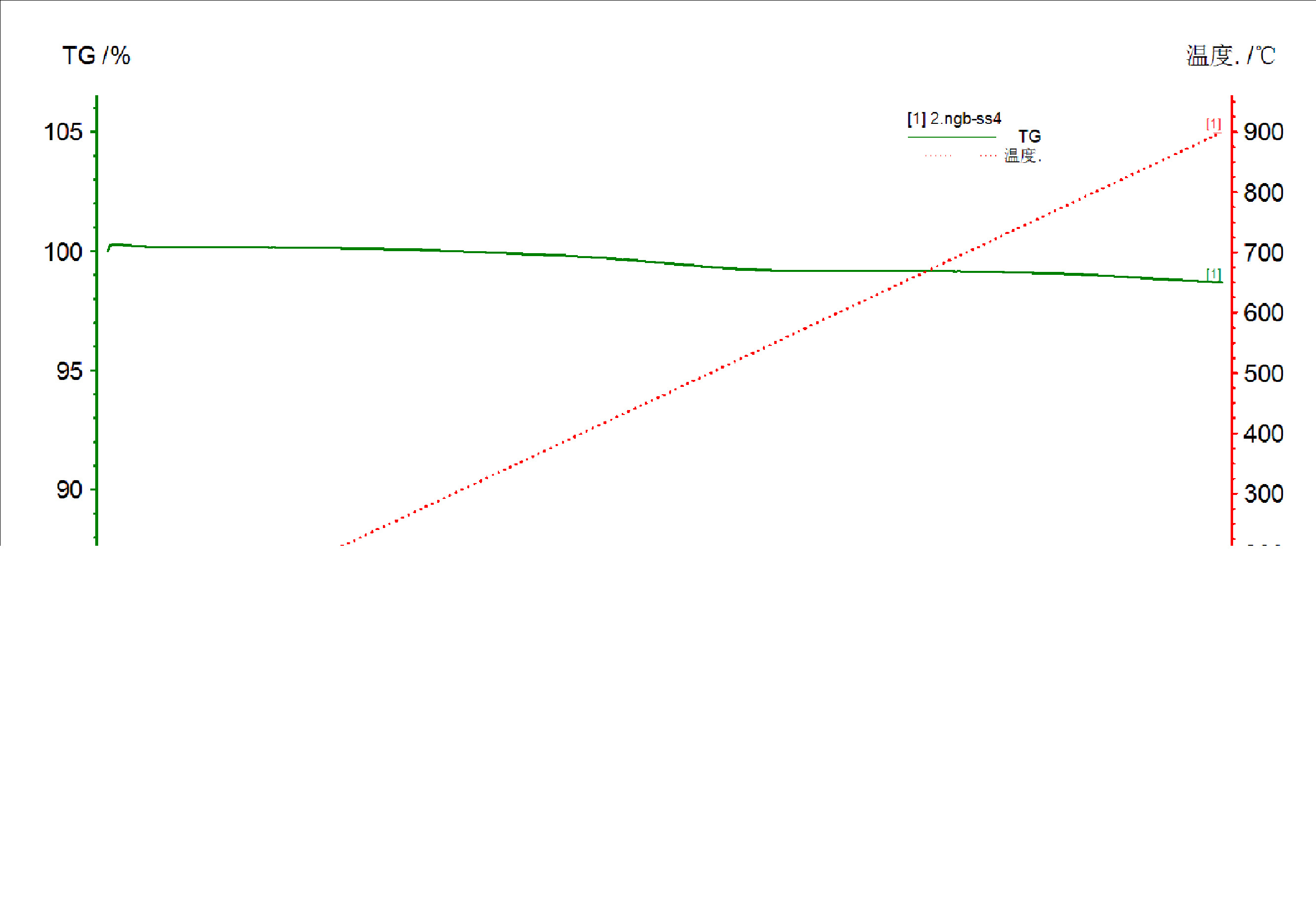

Supplement: Supplementary file 2 [file mmc2.jpg]

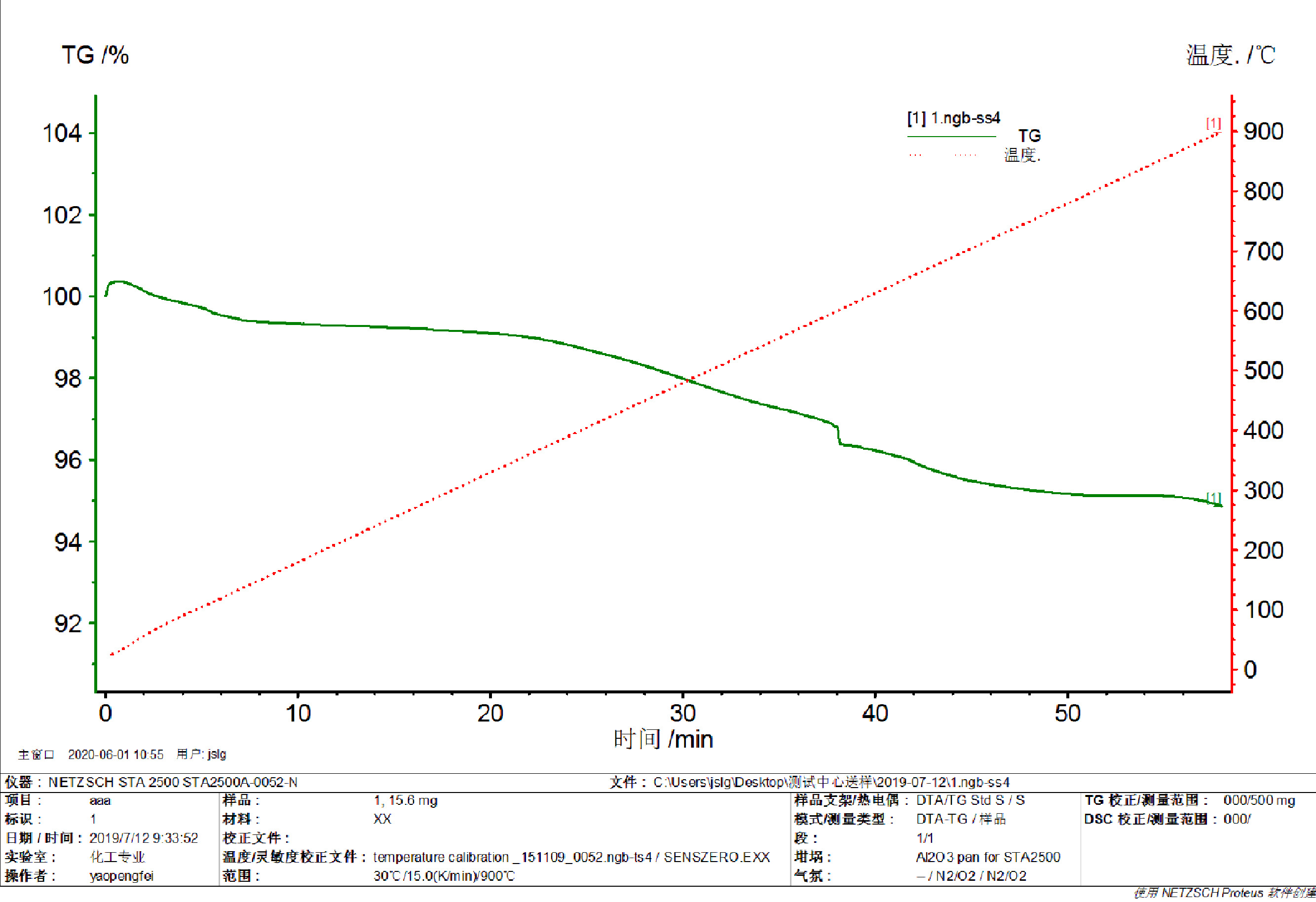

Supplement: Supplementary file 3 [file mmc3.jpg]
